# Energy landscape and dynamics of brain activity during human bistable perception

**DOI:** 10.1038/ncomms5765

**Published:** 2014-08-28

**Authors:** Takamitsu Watanabe, Naoki Masuda, Fukuda Megumi, Ryota Kanai, Geraint Rees

**Affiliations:** 1 Institute of Cognitive Neuroscience, University College London, 17 Queen Square, London WC1N 3AR, UK; 2 Department of Engineering Mathematics, University of Bristol, Woodland Road, Clifton, Bristol BS8 1UB, UK; 3 Centre for Consciousness Science, School of Psychology, University of Sussex, Pevensey 1, Brighton BN1 9QH, UK; 4 Wellcome Trust Centre for Neuroimaging, University College London, 12 Queen Square, London WC1N 3BG, UK

## Abstract

Individual differences in the structure of parietal and prefrontal cortex predict the stability of bistable visual perception. However, the mechanisms linking such individual differences in brain structures to behaviour remain elusive. Here we demonstrate a systematic relationship between the dynamics of brain activity, cortical structure and behaviour underpinning bistable perception. Using fMRI in humans, we find that the activity dynamics during bistable perception are well described as fluctuating between three spatially distributed energy minimums: visual-area-dominant, frontal-area-dominant and intermediate states. Transitions between these energy minimums predicted behaviour, with participants whose brain activity tend to reflect the visual-area-dominant state exhibiting more stable perception and those whose activity transits to frontal-area-dominant states reporting more frequent perceptual switches. Critically, these brain activity dynamics are correlated with individual differences in grey matter volume of the corresponding brain areas. Thus, individual differences in the large-scale dynamics of brain activity link focal brain structure with bistable perception.

## Abstract (web summary)

Bistable visual perception requires changes in brain activity between different cortical areas. Here, Watanabe *et al.* demonstrate dynamic patterns of brain activity during bistable visual perception, which link behavioural variability and anatomical individual differences in focal brain regions.

## Graphical abstract

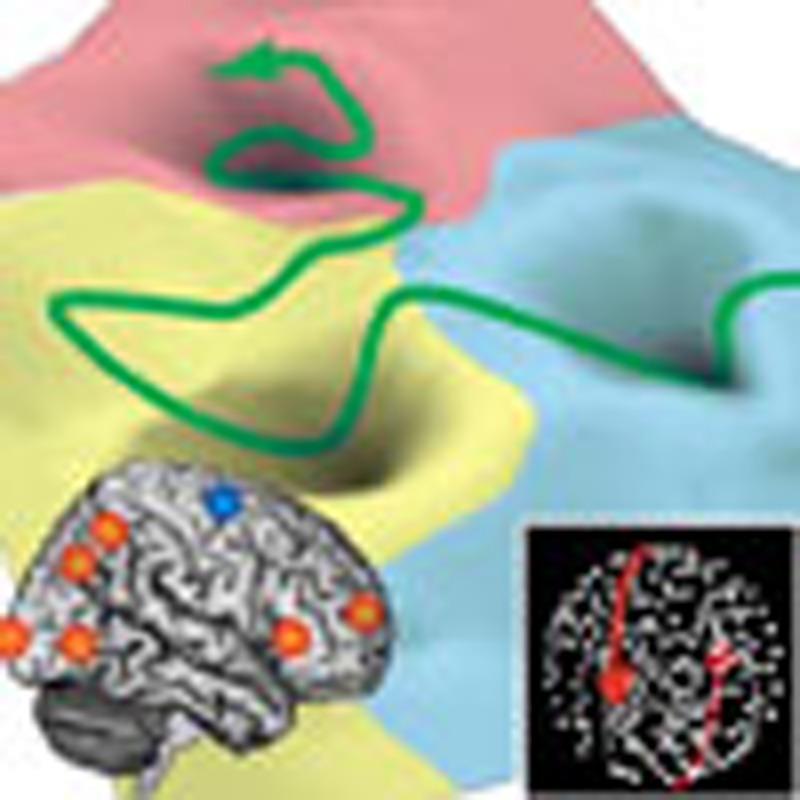

When an ambiguous stimulus is viewed, perception alternates spontaneously between two (or more) perceptual interpretations^1^. In humans, activity throughout the visual system reflects such multistable perception^2^,^3^,^4^,^5^,^6^,^7^,^8^; in addition, parietal and prefrontal cortices are consistently activated in a manner time-locked to the perceptual alternations^9^,^10^,^11^,^12^,^13^. Individual differences in the temporal dynamics of perceptual alternations have been known for over a century^14^,^15^, but more recently such differences have been linked to the structure of either visual cortices^16^ or focal regions of superior parietal lobe^17^,^18^. Currently, however, little is known about the functional mechanisms linking these anatomical features of individual brains to behavioural variability.

Here, to find such links, we estimated the energy landscape of human brain activity using functional magnetic resonance imaging (fMRI) while participants viewed a bistable structure-from-motion (SFM) stimulus (Fig. 1a). We investigated whether there were any systematic relationships between the observed energy landscape, behaviour and participants’ brain structure. Calculation of the energy landscape was motivated by a line of previous results showing that neural activity during multistable behaviours can be described as a series of stays and transitions between different attractors on the energy landscape^19^,^20^,^21^, and such dynamics are influenced by anatomical features in the brain^22^. Considering these observations collectively, we hypothesized that individual features of the dynamics of brain activity on the energy landscape, such as transition rates among attractors, may explain both behaviour reports during bistable perception and the structural characteristics of activated brain regions, thereby bridging the behaviour–structure gap.

We calculated the energy landscape by fitting a pairwise maximum entropy model (MEM)^23^,^24^,^25^,^26^ to brain activity measured using fMRI (see Methods and Supplementary Fig. 1) in seven anatomically defined regions of interest (ROI; Fig. 1a and Supplementary Table 1), whose involvement in bistable perception was repeatedly shown in previous studies^2^,^9^,^12^,^17^,^18^,^27^,^28^. We then numerically simulated dynamical trajectories of brain activity on the energy landscape for each participant and determined the transition pathway of the brain activity that predicted each individual’s pattern of behavioural reports during bistable perception. The obtained energy landscape was compared with that for a ‘replay’ session, where participants viewed non-rivalrous changes in a matched visual stimulus with comparable temporal dynamics. Finally, we investigated whether characteristics of the dynamics on the energy landscape during bistable perception were correlated with the local grey matter volumes of the seven ROIs. These analyses show that brain activity during bistable perception is effectively described as transitions among three distinct states; these transition dynamics for each participant are correlated with both individual behavioural variability and the structural characteristics of focal cortical regions. These results suggest that the features of the energy landscape could link individual characteristics in brain anatomy and difference in subjective visual experience.

## Results

### Local energy minimums during bistable perception

**Figure 1:**
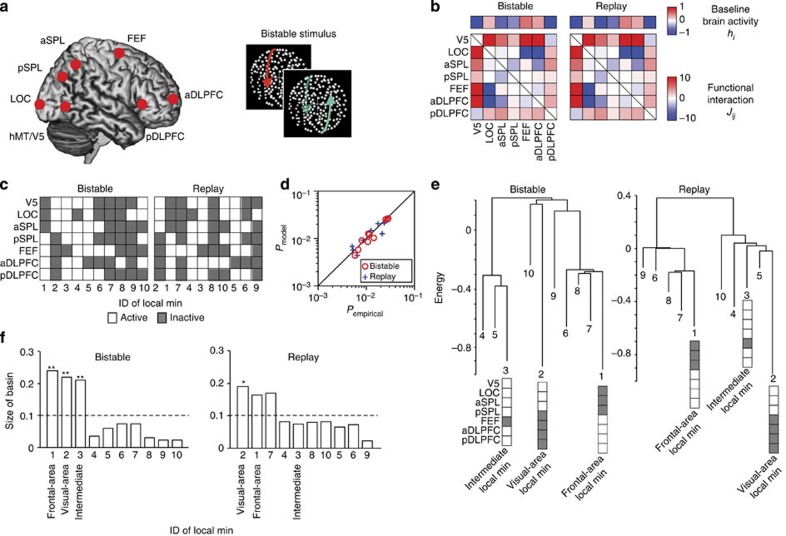
Local minimums in the energy landscape during bistable perception. (**a**) Regions of interest (ROIs) and stimuli. We analysed fMRI signals that were recorded from seven ROIs (red circles in the left panel; Supplementary Table 1 for the coordinates and abbreviations) while participants reported changes in perception associated with a bistable visual stimulus (SFM, right panel). (**b**) Baseline brain activity and functional interactions. The baseline brain activity represents local activity when the given region is isolated, and functional interaction represents pairwise interactions among the brain activity of the seven ROIs. (**c**) Local minimums. The local minimums in each landscape were sorted in an ascending order of energy. Both of them coincidentally consisted of the same ten activity patterns, although the orders differed. (**d**) Comparison of the occurrence probability of the local minimums. The occurrence probability of the local minimums in the empirical data was accurately reproduced by the pairwise MEM with small relative errors (13/17% in bistable/replay session), which was calculated as the ratio of the absolute value of the difference between the predicted and empirical probabilities to the empirical probability. (**e**) Disconnectivity graphs. The graphs show the energy level of the ten local minimums and energy barriers among them. The number attached to each leaf labels the local minimum in **c**. The energy landscape during the bistable session consisted of three major branches that were separated by relatively high-energy barriers and were represented by three local minimums (local min 1, 2 and 3). The local min 1 and 2 also constituted major branches in the energy landscape during the replay session, but the local min 3 did not. (**f**) Size of basin. The size is represented by a proportion of the number of activity patterns belonging to a given basin. The two major local minimums (Frontal-area and Visual-area) had significantly larger sizes of basins than the chance level (0.1) in both the bistable and replay sessions, but Intermediate local min did only in the bistable session. *P<0.05; **P<0.01 in a residual analysis following a chi-square test (degree of freedom=9).

If dynamic brain activity during bistable perception can be described as transitions among relatively stable activity patterns, such stable brain statuses may correspond to local energy minimums. To search for the local energy minimums, we first calculated energy values of all the possible activity patterns of a network consisting of the seven ROIs using the pairwise MEM (Supplementary Fig. 1). As activity at each ROI was binary (active/inactive) in this model, we needed to compare energy values of 2^7^ activity patterns to identify local energy minimums. Technically, the model was fitted to binarized brain activity, and baseline activity of each ROI (*h*_*i*_ in Fig. 1b) and functional interactions among them (*J*_*ij*_ in Fig. 1b) were inferred. We used these parameters to calculate energy values of each activity pattern, and then activity patterns that were locally stable in the sense that the energy value was smaller than those of all their neighbouring patterns. We fitted the pairwise MEM and identified the local minimums on the basis of group-level brain activity and separately for the bistable and replay sessions.

For both bistable and replay sessions, the pairwise MEMs were fitted to the empirical data with high accuracy (86/85% for bistable perception/replay sessions; Supplementary Fig. 2) and gave us information about baseline activity and functional interactions among the ROIs (Fig. 1b). Based on these estimated parameter values, we calculated energy landscapes and found that both bistable perception and replay sessions independently shared the same ten local minimums (Fig. 1c). The estimation of local minimums was accurate in that the empirical probabilities that local energy minimums were visited were close to those inferred by the pairwise MEM (relative error of the observation frequency: 13/17% for bistable perception/replay sessions; Fig. 1d).

Next we identified major local minimums by visualizing hierarchal relationships among the local energy minimums as disconnectivity graphs^29^ (Fig. 1e). By definition, the disconnectivity graph was constructed based on information about energy landscapes, such as the height of energy barriers between local minimums. As the present model implicitly sets the so-called temperature parameter at 1, local minimums separated by energy barriers of the order of <1 should be aggregated. When such a low barrier was observed between neighbouring local minimums, we represented the set of local minimums by the one with the lowest energy among them. As a result of this coarse graining, the local minimums for the bistable perception session were reduced to three local minimums (that is, local min 1, 2 and 3; left panel in Fig. 1e), whereas those for the replay session were reduced to two local minimums (that is, local min 1 and 2; right panel in Fig. 1e). Hereafter, we refer to these representative local minimums as major local minimums for each session. Based on spatial distributions of active regions in each activity pattern, the three major local minimums 1, 2 and 3 are labelled ‘Frontal-area local min’, ‘Visual-area local min’ and ‘Intermediate local min’, respectively.

Notably, these major local minimums had large basins of attraction (Fig. 1f). The size of a basin roughly corresponds to the time spent near the local minimum. During bistable perception, ~70% of activity patterns were included in the basin of one of the three major local minimums (left panel in Fig. 1f). The three basin sizes were significantly larger than the chance level (*χ*^2^(9)=251, *P*<0.001 in a *χ*^2^-test and *z*>2.4, *P*<0.01 in the *post-hoc* residual analysis). During the replay session, brain activity lingered around Frontal-area local min, Visual-area local min and local min 7 (right panel in Fig. 1f). As the energy barrier between Frontal-area local min and local min 7 was much smaller than 1 (right panel in Fig. 1e), activity patterns during the replay session is thought to be represented by the two major local minimums, that is, Frontal-area and Visual-area states.

These results suggest that fluctuation of the brain activity during bistable perception can be efficiently described as dynamic transitions among three major brain statuses represented by Frontal-area, Visual-area and Intermediate local minimums. In particular, the state represented by Intermediate local min may play a crucial role because it was major during bistable perception but not during the replay session.

### Comparison between basin size and behaviour

**Figure 2:**
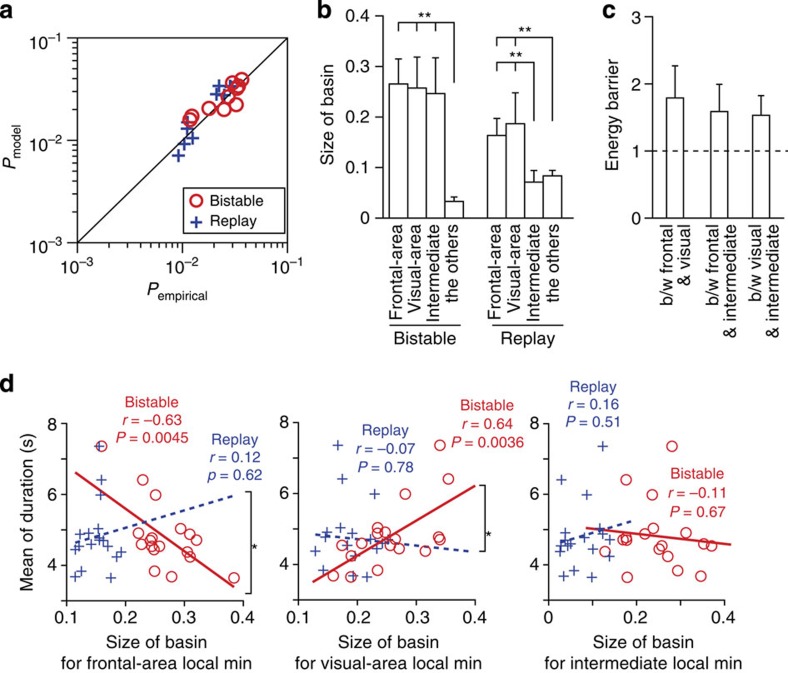
Relationship between basin size and mean duration. (**a**) Comparison of the occurrence probability of the local minimums in single-participant-level analysis. Even for single participants, the pairwise MEM accurately reproduced the occurrence probability of the local minimums in the empirical data. The scatter plot shows the case of participant 1 and the averaged relative error was 17/22% in the bistable/replay sessions, respectively. (**b**) Size of basin. Similar to the sizes of basins observed in the group analysis (Fig. 1f), the three major local minimums (Frontal-area, Visual-area and Intermediate) had significantly larger sizes of basins than the average basin size of the other local minimums in the bistable session. In contrast, the size of Intermediate local min was not large in the replay session. **P<0.01 in signed-rank Wilcoxon tests (*N*=18). Error bars represent the s.d. (**c**) Height of the energy barriers. Similar to the findings of the group analysis (Fig. 1e), in the bistable session the three major local minimums were separated by energy barriers higher than unity. (**d**) Relationship between the size of basin and duration. We compared the basin sizes of the three major local minimums with the mean duration of perception across participants. The subject with a larger basin of local min 1 tended to show shorter duration (that is, more frequent switches), whereas those with a smaller basin of Visual-area local min tended to have more stable perception. The basin size of Intermediate local min had no significant correlation with duration. *Significant difference in correlation coefficients (*r*) between the bistable and replay sessions (*P*<0.05, *N*=18). Red circles and blue crosses represent data for each participant in the bistable and replay sessions, respectively.

We next compared the basin size for each of the three local minimums with behaviour across participants. Before this across-participant analysis, we confirmed that an energy landscape estimated for each participant was qualitatively the same as those estimated from the grouped data. First, even at a single-participant level, the pairwise MEM was fitted to the fMRI data with a high accuracy (83.3±1.8/82.6±1.4% for the bistable perception/replay sessions, mean±s.d. over participants). In addition, the empirical occurrence probability of the individual local minimums was close to the probability inferred by the model (for example, for participant 1, relative error was 17/22% in the bistable perception/replay sessions; Fig. 2a). Furthermore, in all the participants, energy landscapes during bistable perception had the same three major local minimums, which had significantly larger basins than the others (*z*>3.7, *P*_Bonferroni_<0.01 in Wilcoxon signed-rank tests; left panel in Fig. 2b) and were separated by relatively high-energy barriers (minimum height of all the barriers=1.25; Fig. 2c). Finally, consistent with the group-level analysis, the basin of Intermediate local min was significantly smaller than those of Frontal-area and Visual-area local minimums (*z*>3.6, *P*_Bonferroni_<0.01 in Wilcoxon signed-rank; right panel in Fig. 2b), which suggests that Intermediate local min was not major in the replay session.

Given this consistency between group-level and participant-level findings, we then compared the basin sizes of the major local minimums with the mean duration of each alternative percept, which is defined as inter-reversal time spans between spontaneous perceptual switches. We found that mean duration was negatively correlated with the basin size of Frontal-area local min (*r*=–0.63, *P*_Bonferroni_<0.05; left panel in Fig. 2d) and positively correlated with that of Visual-area local min (*r*=0.64, *P*_Bonferroni_<0.05; middle panel in Fig. 2d). These correlations were specific to bistable perception and not observed in the replay session (*z*>2.2, *P*_Bonferroni_<0.05; left and middle panels in Fig. 2d). The basin size of Intermediate local min did not show a significant correlation in either of the two sessions (right panel in Fig. 2d).

These findings show that staying in the basin of Visual-area local min was associated with stabilization of visual perception on one of the two bistable alternatives. In contrast, staying in the basin of Frontal-area local min was associated with switches of visual perception. Intermediate local min, which is approximately a union of Frontal-area and Visual-area local minimums, may represent a brain state associated with transitions between the two local minimums.

### Numerical simulated dynamics of brain activity

**Figure 3:**
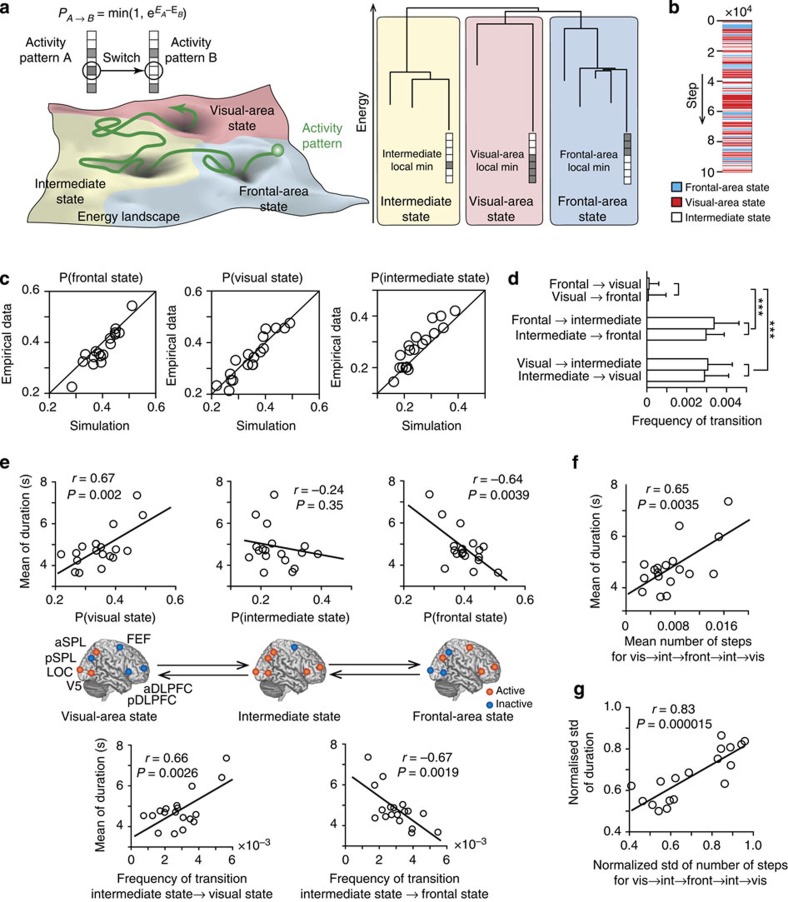
Simulation of dynamics of the brain activity pattern on energy landscapes. (**a**) Schema of numerically simulated dynamics of brain activity. We simulated trajectory of random walk of brain activity pattern on the energy surface for each subject (left panel). The brain activity transits from one pattern to another randomly selected neighbouring pattern. The transition tends to occur along the direction in which the energy decreases (see the upper equation). Based on the disconnectivity graph (right panel), all the activity patterns were classified into one of the three major brain states (Supplementary Fig. 3). (**b**) A sample trajectory of random walk. The colour bar shows the brain states that were simulated on the basis of the data of subject 1. (**c**) Comparison of occurrence probability between the numerical and empirical data. The numerical simulation accurately reproduced the occurrence probability of each brain state in the empirical data with small relative errors (≤9.7%). Each circle represents a participant. (**d**) Frequency of transition between the three major states. Compared with the other transitions, the direct transitions between Frontal-area and Visual-area states rarely occurred (≤3.5% of the others). Error bars: s.d. ***P_Bonferroni_<0.001 in *post-hoc* two-sample *t*-tests (degree of freedom=34). (**e**) Comparison of the numerical results with the behavioural tendency. The probability of Visual-area state and frequency of transition from Intermediate to Visual-area states showed a significant positive correlation with the mean duration, whereas the probability of Frontal-area state and frequency of transition from Intermediate to Frontal-area states showed a negative correlation. (**f**,**g**) Relationship between behaviour and transitions between Visual-area and Frontal-area states via Intermediate state. For the subject with a longer mean duration, the larger number of steps was required for activity pattern to go back and forth between Visual-area and Frontal-area states via Intermediate state. The normalized s.d. of duration was highly predicted by that of the number of steps for the ‘Visual→Intermediate→Frontal→Intermediate→Visual’ transition. We normalized the deviation by dividing it by the mean of the duration for each participant, because the s.d. was highly correlated with the mean.

To further test whether the shape of the energy landscape observed in each participant was consistent with behaviour in the manner observed above, we next numerically simulated dynamics of the brain activity pattern on the observed energy landscapes during bistable perception for each participant using a Markov chain Monte Carlo method with the Metropolis–Hastings algorithm (left panel in Fig. 3a)^30^,^31^,^32^. In this random-walk method, a given activity pattern *A* can transit to another pattern *B* that is randomly chosen from *A*’s neighbouring patterns, that is, those in which the activity (active or inactive) differs from pattern *A* in only one region (upper panel in Fig. 3a). The transition probability is determined by energy values of patterns *A* and *B* (see an equation in Fig. 3a). For each participant, we repeated the random walk 10^5^ steps with different random initial patterns and traced the trajectories of the resulting activity patterns.

The trajectories (i.e., a series of hypothetical activity patterns) were then reduced to a time series of stays and transitions among the three major states represented by the three major local minimums (left panel in Fig. 3a). A major brain state was defined as the set of activity patterns belonging to the basin of a local minimum that was aggregated to one of the three major local minimums (see Supplementary Fig. 3 for the reduction procedure). This reduction was possible for all the participants because, in each participant, any brain activity pattern belonged to a basin of a local minimum, any of which, based on a disconnectivity graph, could be classified into either of the three branches representing a major state (right panel in Fig. 3a). For example, Fig. 3b shows the trajectory for participant 1 (see Supplementary Fig. 4 for the trajectories for the other participants). We confirmed the accuracy of this numerical simulation by finding that the simulation reproduced the occurrence probability of each major brain state in the empirical data with small errors (relative error ≤9.5%; Fig. 3c).

The numerical simulations revealed that the transitions between the Frontal-area and Visual-area states were less frequent than the others (*F*_2,102_=122, *P*<0.001 in a main effect in two-way analysis of variance (three pairs of states × two types of transit direction); *t*_70_>15, *P*_Bonferroni_<0.001 in *post-hoc* two-sample *t*-tests; Fig. 3d). In fact, the frequency of transition between Frontal-area and Visual-area was <3.5% of those between Frontal-area and Intermediate, and between Visual-area and Intermediate, which suggests that most transitions of the brain states during bistable perception occur along the path between Frontal-area and Visual-area states via Intermediate state (middle panel in Fig. 3e).

Next we investigated this Frontal–Intermediate–Visual path by comparing the simulated dynamics with behavioural data (Fig. 3e). Consistent with the empirical data (Fig. 2d), the simulated sojourn probability in Visual-area state was positively correlated with the mean duration of a perceptual state reported behaviourally (*r*=0.67, *P*_Bonferroni_<0.05; upper left panel in Fig. 3e), whereas that in Frontal-area state was negatively correlated with the mean duration of a perceptual state (*r*=–0.64, *P*_Bonferroni_<0.05; upper right panel in Fig. 3e). The sojourn probability in Intermediate state did not show any significant correlation with behaviour (*r*=–0.24, *P*=0.35; upper middle panel in Fig. 3e). In addition, the transition frequency from Intermediate to Visual-area states was positively correlated with the mean duration (*r*=0.66, *P*_Bonferroni_<0.05; lower left panel in Fig. 3e), and that from Intermediate to Frontal-area states had a negative correlation (*r*=–0.67, *P*_Bonferroni_<0.05; lower right panel in Fig. 3e). None of the other transition frequencies showed any such significant correlations (see Supplementary Fig. 5).

These findings support the notion that during bistable perception, brain states fluctuated between Frontal-area and Visual-area states via Intermediate state; staying in Frontal-area state induced a perceptual switch, while staying in Visual-area state stabilized perception.

We obtained further evidence for this possibility through direct comparison of the empirically observed behaviour during bistable perception with the simulated dynamics confined to the Frontal–Intermediate–Visual path. The mean duration of bistable percept was strongly correlated with the average of the simulated time that was required for one return trip between Visual-area and Frontal-area states via Intermediate state (that is, Visual→Intermediate→Frontal→Intermediate→Visual; *r*=0.65, *P*=0.0035; Fig. 3f). Moreover, the normalized s.d. of duration was also predicted by that of the time for the return trip (*r*=0.83, *P*=0.000015; Fig. 3g).

### Relationship between energy landscape and brain structure

**Figure 4:**
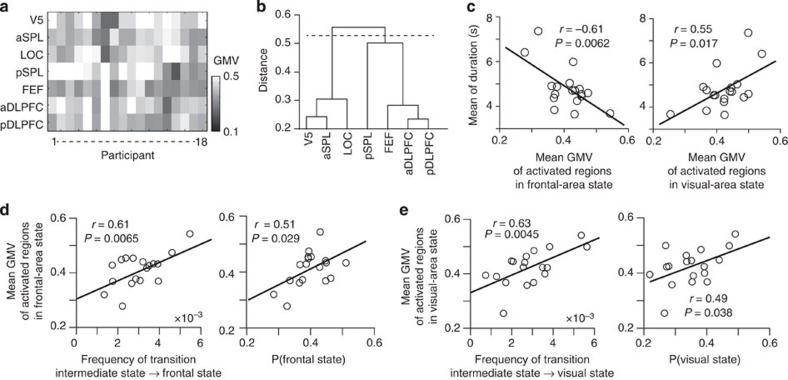
Relationship with GMV. (**a**,**b**) Hierarchical clustering of the seven ROIs based on their GMV. On the basis of the GMV (**a**), we performed a hierarchical clustering (**b**). Consistent with the findings about the energy landscape, the seven ROIs were classified into the two groups, corresponding to Frontal-area and Visual-area states derived from the energy landscape, respectively. The abbreviations are the same as in Fig. 1a. (**c**) Correlation between GMV and duration. As partly shown in previous studies, mean duration of bistable perception was significantly correlated with the mean GVM of activated regions in each energy state (that is, pSPL, FEF, aDLPFC and pDLPFC for Frontal-area state; V5, LOC and aSPL for Visual-area state). Each circle represents a participant. (**d**,**e**) Relationship between GMV and energy landscape. Participants (circles) with high frequency of transition from Intermediate to Frontal-area states and high probability of Frontal-area state tended to have large GMVs of the regions activated in Frontal-area state. In contrast, subjects with high frequency from Intermediate to Visual-area states and high probability of Visual-area states tended to have large GMVs of the regions activated in Visual-area state.

Brain activity dynamics on the energy landscape can reflect anatomical features of the brain^22^. Therefore, we conjectured that the properties of individual energy landscapes may also be associated with individual differences in the anatomical brain structures that are known to be relevant to subjective experience of bistable perception^15^,^17^,^18^,^33^. For example, a participant whose brain state tends to stay in Frontal-area state may have larger grey matter values (GMVs) in the brain regions activated in Frontal-area state. We tested this hypothesis by comparing dynamical properties of activity patterns with GMVs of the seven ROIs across participants (Fig. 4a).

We first examined whether GMV-based classification of the ROIs could reproduce that based on the pairwise MEM into the two major states (i.e., Frontal-area and Visual-area states). To this end, we performed a hierarchal clustering of the ROIs, in which the regions were classified based on the similarity of inter-individual variability of their GMVs. Remarkably, the hierarchal clustering divided the seven ROIs into the same two groups corresponding to these two major states (Fig. 4b). Moreover, the average GMV of each group showed a significant correlation with behaviour (Fig. 4c): the mean GMV of the brain regions activated in Frontal-area state was negatively correlated with the mean duration (*r*=–0.61, *P*=0.0062), whereas that in Visual-area state was positively correlated with behaviour (*r*=0.55, *P*=0.017). These results are consistent with previous studies focusing on GMVs of one of the seven ROIs: the study by Kanai *et al.*^17^ showed a negative correlation between GMV of posterior superior parietal lobule (pSPL), which was activated in the present study as part of Frontal-area state, and mean duration during bistable perception; a second study by Kanai *et al.*^18^ reported a positive correlation between GMV of anterior superior parietal lobule (aSPL), which was activated in Visual-area state, and behaviour; finally, a human neuroimaging study showed that improvement of ability to stably detect motion coherence was associated with an increase in GMV in hMT/V5, which was activated in Visual-area state in the present study^34^.

We then confirmed our hypothesis by revealing statistically significant correlations between these average GMVs and several key features of the energy landscape during bistable perception. The mean GMV for Frontal-area state was well predicted by the transition frequency from Intermediate to Frontal-area states (*r*=0.61, *P*=0.0065) and the sojourn probability in Frontal-area state (*r*=0.51, *P*=0.029; Fig. 4d). Similarly, the GMV for Visual-area state was correlated with the transition frequency from Intermediate to Visual-area states (*r*=0.63, *P*=0.0045) and the sojourn probability in Visual-area state (*r*=0.49, *P*=0.038; Fig. 4e). These correlations provide support for the notion that macroscopic dynamics of brain activity on the energy landscape bridge the gap between the anatomical brain structures and individual differences in behaviour during bistable visual perception.

## Discussion

We found that brain activity patterns during bistable perception can be effectively described as transitions between a visual-area-dominant state and a frontal-area-dominant state via an intermediate state. The tendency of the dynamics for each participant was significantly correlated with both individual behavioural variability during bistable perception and the structural characteristics of focal cortical regions. Although the causality among energy landscape, anatomy and behaviour could not be inferred here, these present findings suggest that the dynamics of brain activity determined by the features of the energy landscape link individual differences in brain anatomy and subjective visual experience.

The dynamics of human brain activity we observed can potentially account for previous findings about the effects of focal lesion and deactivation on bistable perception using an ambiguous figure^17^,^18^,^35^. For example, our data predict that artificial temporal deactivation of aSPL should decrease the sojourn probability in Visual-area state, in which aSPL is active. In contrast, such deactivation should increase the sojourn probability at Frontal-area state, in which aSPL is (relatively) inactive. Such a bias would induce frequent percept switches rather than stable perception, thereby resulting in the decreased duration of percepts observed^17^,^18^. Under the same logic, the present findings also explain a mechanism for why transcranial magnetic stimulation (TMS) applied to pSPL increases perceptual duration for an SFM stimulus^17^. Moreover, lesions to prefrontal cortex reduce switch rates for ambiguous figures but do not affect the effort of maintaining one perception^35^, consistent with the current findings that Frontal-area state is associated with perceptual switches. Thus, our findings and framework can account for a number of key findings in the literature on bistable perception obtained using a range of different techniques.

The current study assumed that an attractor-network model was able to describe the dynamics of brain activity during bistable perception. Energy-based explanations of various multistable behaviours are found in classical theoretical studies^36^. For bistable perception, theoretical models based on stochastic resonance^37^ and in a context of self-organizing patterns in open systems^38^ were employed for explaining behaviour. In the last decade, this framework has been used for interpreting how bistable perception is affected by cumulative perception history^39^,^40^, internal neural noise^41^,^42^, or both^43^. Furthermore, several groups have added experimental evidence in favour of this idea (see reviews refs 19, 21). The present findings add direct experimental evidence to support attractor dynamics in bistable perception but in a novel way. The previous literature mentioned above has assumed that distinctive attractors correspond to different percepts. By contrast, the current findings suggest that brain activity roughly transits between a state related to perceptual stability (that is, Visual-area state) and one related to perceptual transitions (that is, Frontal-area state).

Such an energy-based framework is also complementary to other models of neural activity underlying bistable visual perception, such as those assuming inter-neuronal suppression and competition in the visual area (see reviews refs 44, 45). In fact, the present study focused on the activity of a large-scale brain network rather than on local neuronal activity in, for example, hMT/V5. In addition, the present study does not indicate whether brain activity associated with bistable perception is mainly affected by top-down or bottom-down signals. As shown in Fig. 1b, the estimated pairwise functional interactions do not contain information about their directionality and, therefore, to reveal the causality among the ROIs, further studies using a dynamic causal model (or other forms of model-based inference) are necessary.

The ROIs were selected as brain regions whose activations have been repeatedly reported in neuroimaging studies on bistable perception using an ambiguous figure^2^,^9^,^12^,^17^,^18^,^27^,^28^. The number of ROIs needed to be small to ensure accuracy of the fit of the pairwise MEM given the constraints of the amount of data. In such a model, increased number of ROIs results in the exponential increase number of potential brain activity patterns, and thus the amount of the data necessary for accurate estimation also dramatically increases. Given that the number of the fMRI images recorded during bistable perception for each participant was around 600, 7 was the largest number of ROIs for accurate fit (that is, accuracy of fit >80%). Under this restriction, we attempted to balance the number of ROIs among the three different brain areas. As a result, two ROIs were chosen from the visual and parietal areas, and three ROIs were from the frontal areas. Although there is a possibility that different energy landscapes might be observed when the model uses a larger number or different sets of ROIs, the present study added evidence for the importance of these seven ROIs in bistable visual perception.

The high accuracy of the current parsimonious model based on only seven ROIs might also be attributed to the fact that all the ROIs belong to major large-scale brain networks: hMT/V5 and lateral occipital complex (LOC) belong to the visual network, while aSPL, pSPL, frontal eye field (FEF), anterior dorso-lateral prefrontal cortex (aDLPFC) and posterior dorso-lateral prefrontal cortex (pDLPFC) to the front-parietal network^46^. Furthermore, pSPL, aDLPFC and pDLPFC are considered to play roles of hubs in the network^47^,^48^. Because of these network properties, such a model consisting of a few regions might be enable to represent essential parts of whole-brain activity during bistable perception.

In our previous study using resting-state fMRI data^25^, the pairwise MEM-extracted functional interactions may have been biased towards recovering underlying anatomical connections. Therefore, the energy landscapes estimated in the current study may be also biased to underlying structural anatomy rather than to task-related patterns of connectivity. However, here we observed striking and statistically distinguishable differences in the features of the different energy landscapes comparing the two different task conditions (that is, bistable and replay sessions; Figs 1c,e,f and 2b,d). Moreover, the energy landscape during fixation periods also showed largely different characteristics (Supplementary Fig. 6) to those obtained during fixation. The energy landscape during the fixation periods consisted of three local minimums, none of which were among the three major local minimums found during bistable perception sessions. The energy landscape was rather similar to that found in the fronto-parietal network during resting state^49^, which is consistent with the similarity between fixation periods and resting state. As a whole, these observations support the task specificity of the energy landscape extracted by pairwise MEM, and imply that although anatomical structures might underlie such cognitive processes, energy landscape is mainly affected by task-specific activity.

In addition, we should note that the present findings do not indicate any causal relationships among energy landscapes, brain anatomy and behaviour. A line of studies have accumulated evidence relating the morphology of individual brain regions to performance on various neuropsychological tasks ranging from basic cognitive functions such as perception^50^,^51^ and memory^52^ to complex and high-order functions, including literacy^53^ and social activity^54^,^55^. Furthermore, the strength of some of these structure–behaviour relationships has a positive correlation with the length of training and learning^52^,^56^,^57^. However, it remains under debate whether these anatomical changes are the cause or the consequence of the specific behaviours and their repetition^33^,^58^. We propose that the features of the energy landscape during bistable perception are likely to constitute intermediate endophenotypes between the anatomical variety and the behavioural difference. That is, the energy landscape plays a role of media and allows interactions between the anatomical features and behavioural tendency: repetition of a specific visual percept experience may induce bias in the energy landscape, which may consequently cause anatomical change; hereditary anatomical difference may induce and enhance the features in the energy landscape, thereby resulting in the biased behaviour. To clarify the direction of causality among these factors, further studies such as longitudinal observations are necessary.

The present study has demonstrated that characteristics of the energy landscapes of the brain activity can link anatomical differences to behavioural inter-individual variability. We hope that this finding will lead us to more integrative understanding of the neural mechanisms underlying dynamics of consciousness.

## Methods

### Participants

Behavioural and MRI data were recorded from 18 healthy right-handed participants with normal vision (9 females, age: 21–40 years). Written informed consent was obtained from all participants and the experiments were approved by the UCL Research Ethics Committee.

### Stimuli

In both the bistable perception blocks and the replay blocks, stimuli were presented on the screen mounted on the MRI head coil using a JVC DLA-SX21 projector (screen size: 27 cm × 21 cm; spatial resolution: 1,024 × 768) through a mirror attached to the coil. Viewing distance was ~72 cm. For dichoptic viewing, prism glasses (lenses with four prism dioptres base out) and a black cardboard attached to the head coil were used^59^. The cardboard divided the screen and mirror into the right and left visual hemi-fields without overlapping.

In the bistable perception blocks, two identical vertically spinning spheres were presented to the two eyes of the participants. The spheres were created using PsychToolbox 3 in MATLAB (Mathworks) and consisted of 200 full-contrast white dots subtending 3.1° diameter on black background^17^. A fixation cross (0.1° in height and width) was superimposed at the centre of the spheres (Fig. 1a). The white dots moved sinusoidally up and down at an angular velocity of 120° s^−1^. A square surrounded the spheres stimuli for stable vergence.

In the replay trials, binocular disparity was calculated for each dot so that stimuli had apparent disparity cues and the participants easily perceived stereoscopic depth. The fixation points and rectangles were aligned to the centre of the spheres as in the bistable perception session.

### Experiment design

In the MRI scanner, participants viewed seven runs of three bistable perception blocks and seven runs of three replay blocks. In the bistable perception blocks, they were presented with the above-mentioned SFM stimulus, which induces spontaneous switches of subjective visual perception. In the replay blocks, they were presented with a series of slightly different images to each eye and experienced stimulus-driven change of their perception. In this condition, the percept experience reported during the bistable condition was replayed. The order of rivalry block and replay block was pseudo-randomized across runs and participants.

In both conditions, each block consisted of a stimulus period (31.5 s) and a following fixation period (11 s). Although a continuous long single-block design (for example, 15 min per block) may be theoretically suitable for the following data analysis, we adopted this multiple block design to make it easier for participants to keep their concentration during the task and to prevent them from falling asleep during the passive perception task. The participants were instructed to look at stimuli through prism glasses and report their subjective perception (that is, the direction of rotation of the sphere) by pressing one of three buttons (one for a sphere rotating towards the participant, another for a sphere rotating away from the participant and the other for uncertain perception). In the following neuroimaging analysis, we did not exclude fMRI signals during the uncertain perception, whose length was 2.2±0.1% (mean±s.d.) of that of the entire bistable perception sessions. It is because our goal is to describe the entire dynamics of brain activity during the whole bistable perception sessions without using behavioural information. Consistent with this, we did not use in the data analysis the information about when the button for uncertain perception was pressed. That is, percept duration was calculated as an interval between time points at which participants pressed the button to indicate switches of their perception.

Before the MRI scanning, we performed behavioural tests on the participants and confirmed that they could experience spontaneous percept switches in a suitable time range (3 to 10 s) in our experiment setup.

### Data acquisition

Images were obtained using a 3T MRI (Magnetom Trio, Siemens) with the 32-channel head coil at the Wellcome Trust Centre for Neuroimaging at University College London. Functional images were recorded using an echo planar imaging (EPI) sequence (repetition time, 2.1 s; echo time, 30 ms; 30 axial slices; 3 mm isotopic; Field of view (FOV), 192 × 192 mm). T1-weighted structural images were acquired with spatial resolution of 1 × 1 × 1 mm. Phase image and magnitude images were also obtained to compute a fieldmap. These stimuli and methods for data acquisition are the same as our different study (Megumi F. *et al.*, in preparation).

### fMRI data preprocessing

The functional images were preprocessed using SPM8 ( http://www.fil.ion.ucl.ac.uk/spm). The first five EPI volumes were discarded to allow for T1 equilibration. The EPI images were realigned, unwrapped based on fieldmap images using the FieldMap toolbox, corrected for slice timing, normalized to the Montreal Neurological Institute (MNI) stereotactic template and spatially smoothed (Gaussian kernel: 8 mm full-width at half-maximum).

Owing to slow and long-lasting haemodynamic reactions, fMRI images recorded at adjacent time points are strongly correlated, which can adversely affect the MEM analysis. As in our previous study^26^, we reduced the correlation by applying a general linear model to these data as follows: if the data for a given subject consisted of *T* images, we built *T* regressors whose onset was set to the start time of each image acquisition. After convolving the regressors with a haemodynamic response function implemented in SPM8, we estimated regression coefficients, which are thought to represent brain activity at each time point of data acquisition. Six head-motion parameters and parameters representing each scanning run were also implemented in this general linear model as covariates of no interest.

We then extracted and concatenated fMRI signals during stimulus periods (31.5 s for each period) for each ROI for each participant (Supplementary Fig. 4). The concatenation of fMRI data had little effect on the following analysis because the MEM by definition regarded brain activity patterns at different time points as independent from each other. Temporal information was not used for inferring energy landscapes. It should be noted that this extraction was based on the timing of actual presentation of the stimuli and did not use behavioural information about perception switches during the presentation periods.

The coordinates of the seven ROIs were selected on the basis of a line of previous studies on bistable perception (Supplementary Table 1): the coordinates of hMT/V5 and LOC were based on a study by Freeman *et al.*^2^; those of aSPL and pSPL were the same as those in studies on TMS-induced effects on bistable perception^17^,^18^,^27^; the coordinates of FEF were based on a study by Sterzer *et al.*^12^; those of aDLPFC and pDLPFC were determined by a study by Knapen *et al.*^28^ and one by Kleinschmidt *et al.*^9^, respectively. All the ROIs were defined as spheres of 4 mm radius.

### Fitting of the pairwise MEM

We fit the pairwise MEM to the preprocessed fMRI signals as follows in the same manner as that employed in our previous study^25^,^26^. For each ROI, we first binarized the obtained fMRI signals with a threshold that was defined as the time-averaged activity of the same ROI during fixation periods. Previous studies suggest that binarization does not eliminate important information contained in originally continuous brain signals: electrophysiological studies succeeded in estimating simplicity and topological characteristics of brain networks from binarized local field potential data^60^,^61^, which are highly correlated with fMRI signals^62^; a theoretical study demonstrated that binary brain activity can reproduce functional interactions similar to those measured by fMRI^22^; our previous study also showed that binarized fMRI signals described anatomical connection better than continuous neural signals^25^. Therefore, we expected that we could obtain some biologically meaningful information from binary fMRI signals.

The binarized activity at brain region *i* and discrete time *t*, denoted by 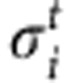, is either active (+1) or inactive (0). The activity pattern at time *t* is described by 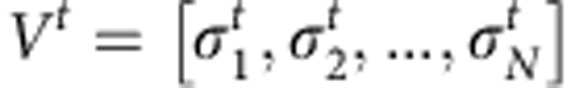, where *N* (=7) is the number of the brain regions. The empirical activation probability of region *i*, ‹*σ*_*i*_›, is equal to 
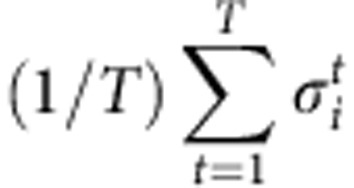
, where *T* is the number of snapshots. The empirical pairwise activation probability of regions *i* and *j*, ‹*σ*_*i*_*σ*_*j*_›, is equal to 
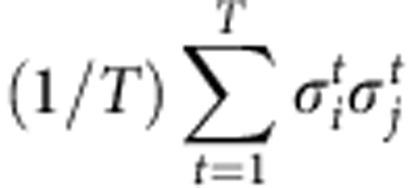
.

Under the restriction that ‹*σ*_*i*_›_m_ and ‹*σ*_*i*_*σ*_*j*_›_m_ (that is, average of *σ*_*i*_ and *σ*_*i*_*σ*_*j*_, respectively) inferred from by the pairwise MEM were equal to the empirical values ‹*σ*_*i*_› and ‹*σ*_*i*_*σ*_*j*_›, respectively, we searched for the probability distribution *P*(*V*_*k*_), where *V*_*k*_ was the *k* th brain activity pattern, which maximizes the entropy. The principle of maximum entropy^63^ states that the probability distribution maximizing uncertainty, corresponding to the most random distribution given the constraints, should be selected. Especially, when ‹*σ*_*i*_› and ‹*σ*_*i*_*σ*_*j*_› are constrained by the data, the probability distribution with the largest entropy is the Boltzmann distribution^63^, that is, 
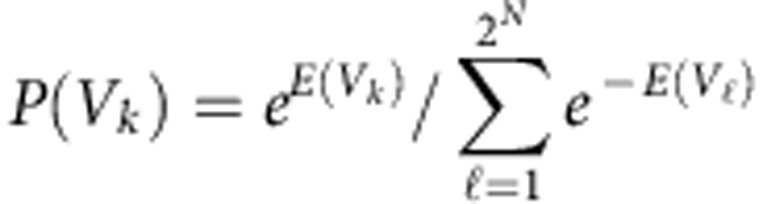
, where *E*(*V*_*k*_) is the energy of activity pattern *V*_*k*_ and is given by 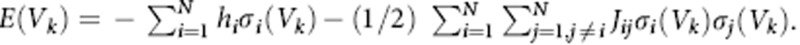 Here, *σ*_*i*_(*V*_*k*_) represents the binarized activity (that is, 0 or 1) at region *i* under activity pattern *V*_*k*_.

Technically, we adjusted *h*_*i*_ and *J*_*ij*_ in the Boltzmann distribution until 
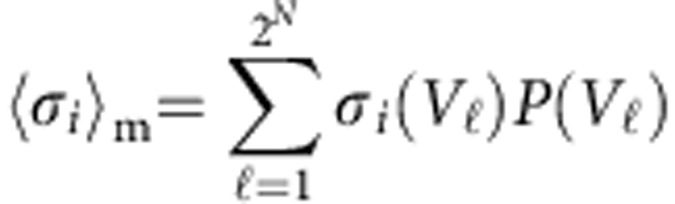
 and 
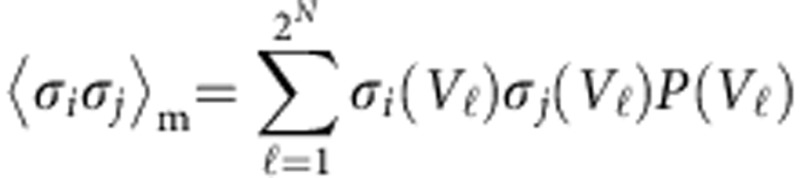
, that is, the averages calculated from the model, are approximately equal to the empirical values ‹*σ*_*i*_› and ‹*σ*_*i*_*σ*_*j*_›. This adjustment of *h*_*i*_ and *J*_*ij*_ was performed based on a gradient ascent algorithm. Precisely speaking, we set 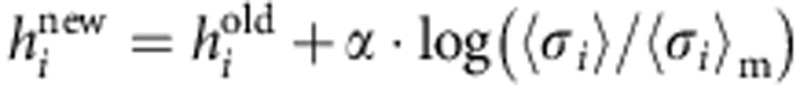 and 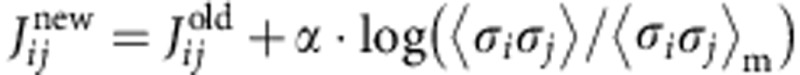 in a single adjustment event, where *α*=0.25. The estimated *h*_*i*_ and *J*_*ij*_ values were robust against moderate variations in *α*. In other words, with all *α*-values ranging from 0.1 to 0.4 in units of 0.05, the estimated *h*_*i*_ and *J*_*ij*_ values were almost the same as those with *α*=0.25 (correlation coefficient>0.92). The calculation of *h*_*i*_ and *J*_*ij*_ was independently performed for data during bistable perception and those during the replay session.

### Energy landscape and local minimums

We calculated the energy landscape in the same way as in our previous study^49^. The energy landscape is defined as a network of brain activity patterns *V*_*k*_ (*k*=1, 2, …, 2^*N*^) with the corresponding energy *E*(*V*_*k*_). Two activity patterns are regarded as adjacent in the network if and only if they take the opposite binary activity at just one brain region (upper panel in Fig. 3a). We first exhaustively searched for local energy minimums, whose energy values are smaller than those of all the *N* adjacent patterns. We then constructed disconnectivity graphs^29^, which mapped out the connectivity among local energy minimums, as follows. First, we started with the so-called hypercube graph, in which each node representing a brain activity pattern is adjacent to the *N* neighbouring nodes. Second, we set a threshold energy level, *E*_th_, at the largest energy value among those for the 2^*N*^ nodes. Third, we removed the nodes whose energy level was equal to or higher than *E*_th_. All links incident to the removed nodes were also deleted. Fourth, we judged whether each pair of local minimums was connected by a path in the reduced network. Fifth, we repeated the third and fourth steps after moving *E*_th_ down to the next largest energy value. We ended up with a reduced network in which each local minimum was isolated. Sixth, based on the obtained results, we built a hierarchical tree whose leaves (that is, terminal nodes down in the tree) were the local minimums. The vertical position of the leaves and internal nodes represents an energy value. An internal node shows the point at which the branching of different groups of local minimums takes place. This hierarchical tree was referred to a disconnectivity graph.

### Basin sizes and energy barriers

We then estimated the basin sizes of local minimums and energy barriers between them as we did in our previous study^49^. To calculate the basin sizes, we first selected a starting node *i* among the 2^*N*^ nodes. Then, if any of its neighbour nodes has a smaller value of energy than node *i*, we moved to the neighbour node with the smallest energy value. Otherwise, we did not move, which implied that node *i* was a local minimum. We repeated this procedure until we arrived at a local minimum. The starting node *i* was regarded to belong to the basin of the local minimum that was finally reached. We calculated the corresponding local minimum for all *i*. The basin size of a local minimum was equal to the fraction of nodes that belonged to the basin of the local minimum.

An energy barrier between a pair of local minimums *i* and *j* was calculated as min[*E*^*b*^(*V*_*i*_,*V*_*j*_)−*V*_*i*_,*E*^*b*^(*V*_*i*_,*V*_*j*_)−*V*_*j*_], where *E*^*b*^(*V*_*i*_,*V*_*j*_) is the threshold energy level at which the disconnectivity graph branches into a group including *i* and a group including *j*. If the energy barrier is high, the transition of brain activity patterns between *i* and *j* occurs at a small rate at least in one direction. Although the transition occurs at different rates in the two directions (that is, from *i* to *j* and from *j* to *i*) if *V*_*i*_ and *V*_*j*_ are different^38^, we used the symmetric definition given above for simplicity^31^.

We separately performed the series of these procedures (that is, fitting of the pairwise MEM, searching for local minimums, constructing a disconnectivity graph and estimating the basin sizes and energy barriers) for grouped and individual data.

### Numerical simulations of dynamics on the energy landscape

Based on the energy landscape estimated for each participant, we numerically simulated dynamics of the brain activity patterns using a Markov chain Monte Carlo method with the Metropolis–Hastings algorithm^30^,^31^,^32^. We allowed any brain activity pattern *V*_*i*_ to move only to a neighbouring pattern *V*_*j*_ that is selected from all the *N* neighbours with the uniform probability 1/*N* (Fig. 3a). The actual transition from *V*_*i*_ to *V*_*j*_ occurred with probability *P*_*ij*_=min[1,*e*^*E*(*Vi*)−*E*(*Vj*)^]. For each participant, we repeated the random walk 10^5^ steps with a randomly chosen initial pattern. We then reduced the trajectory of activity patterns to a series of stays and transitions among the three major states represented by the three local minimums (Frontal-area, Visual-area and Intermediate local minimums). We discarded the first 100 steps as a transient to eradicate the effects of the initial condition.

These methods to investigate energy landscape are comparable to some graph-theoretical measures to detect communities of nodes in complex networks (see review ref. 64): for example, one method uses a dendrogram based on a type of distance that is quantified through Brownian random walk^65^. Another measure defines communities by performing Monte-Carlo-approach random walk on the energy landscape^66^, in which energy value was assigned to each node based on its topological feature^67^.

### Grey matter volume analysis

We first preprocessed the high-resolution T1-weighed images in SPM8 as follows^68^. For each participant, the image was segmented into grey matter (GM), white matter and cerebrospinal fluids in the native space with the New Segment Toolbox^69^. The segmented GM images underwent alignment, warp to a template space and resampling down to 1.5-mm isotropic voxels. We registered the GM images to a subject-specific template using the DARTEL Toolbox^70^. We normalized individual GM images to MNI spaces by using the DARTEL Toolbox and smoothed the images with a Gaussian kernel (full-width at half-maximum=8 mm). After normalizing by the whole-brain values of the GMVs, we extracted the regional GMV for each ROI; the ROI was defined as a 4-mm-raidus sphere whose centre was located at the coordinates shown in Supplementary Table 1 (Fig. 4a).

Using the GMV data, we first performed a hierarchal clustering of the seven ROIs using MATLAB: for each ROI, we first built an 18-dimensional vector consisting of GMVs recorded from 18 participants. We then calculated the Hamming distances between pairs of the vectors and constructed a dendrogram composed of the seven GMV vectors. The dendrogram allowed us to classify the ROIs into two groups. For each participant, we then averaged the GMVs over the ROIs belonging to each group. We finally calculated the Pearson’s correlation coefficients between the averaged GMVs and the individual features of the above-estimated energy landscape such as frequency of transition and probability of stay in each major brain state.

### Statistics

All *post-hoc t*-tests were corrected using a Bonferroni correction for multiple comparisons. In the correlation analysis using the grouped data (Fig. 2d), the statistical threshold for each correlation was set at 0.008 (=0.05/6; three major local min × two types of condition (bistable, replay)). In the case using simulated data (Fig. 3e), we set the threshold at 0.007 (0.05/7; three major states+four types of transitions among the states). It should be noted that the correction was not necessary for the correlation analysis between the GMVs and features of energy landscapes because we performed the analysis based on the explicit hypothesis that the GMVs reflect the characters of the energy surface.

## Additional information

**How to cite this article:** Watanabe, T. *et al.* Energy landscape and dynamics of brain activity during human bistable perception. *Nat. Commun.* 5:4765 doi: 10.1038/ncomms5765 (2014).

## Supplementary Material

Supplementary Information Supplementary Figures 1-6, Supplementary Table 1 and Supplementary References
