# Supplementary material for: Energy landscape and dynamics of brain activity during human bistable perception
Source: Nat Commun. 2014 Aug 28;5:4765. doi: 10.1038/ncomms5765 (PMC4174295; doi:10.1038/ncomms5765)
Supplement: Supplementary Information — Supplementary Figures 1-6, Supplementary Table 1 and Supplementary References [file ncomms5765-s1.pdf]

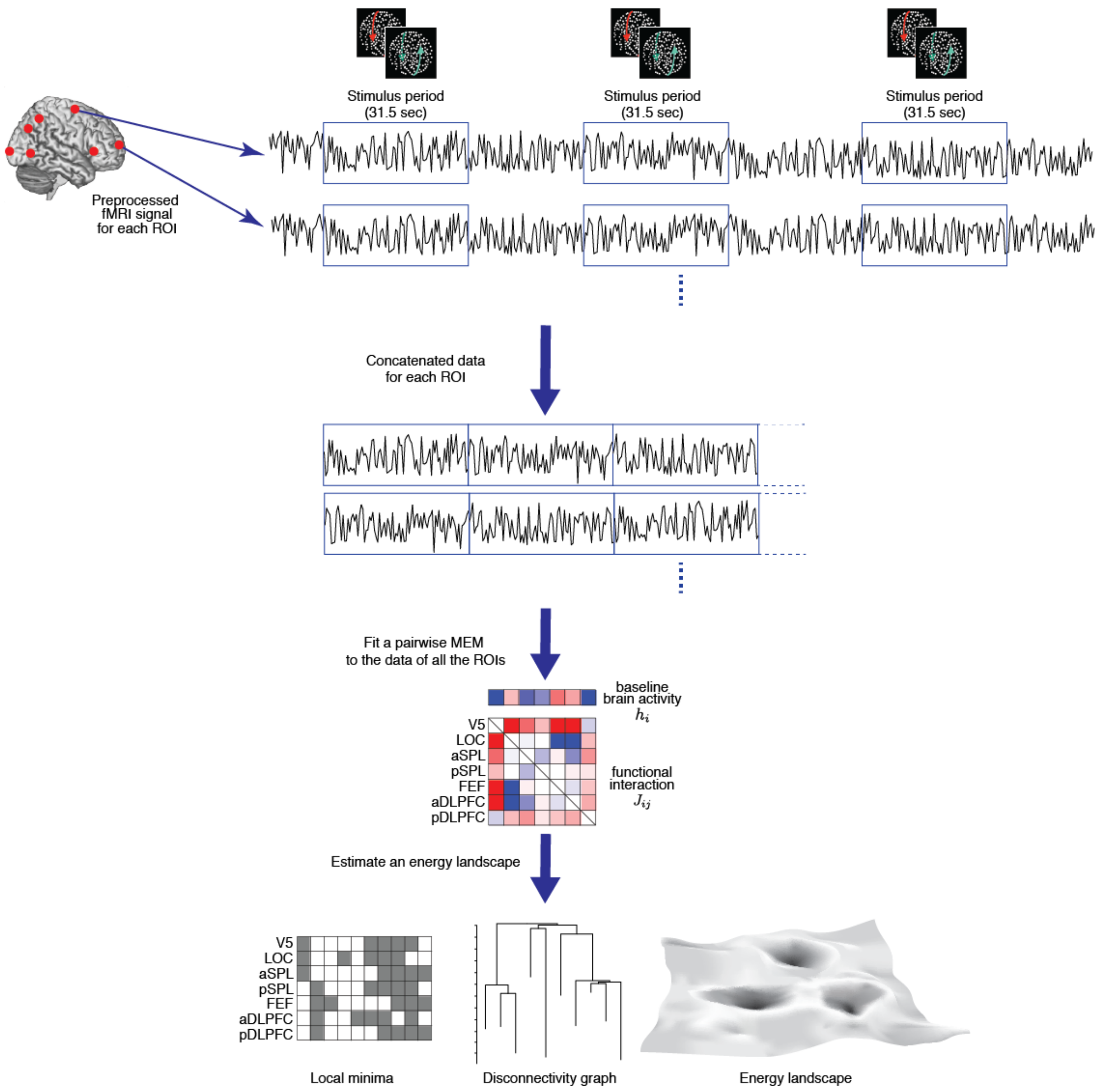

Supplementary Figure 1. Schema of the analysis procedure for estimation of energy landscapes.

After preprocessing, the fMRI data during stimulus periods were extracted and concatenated for each ROI. We then fitted a pairwise MEM to the data collected from all the ROIs, and calculated the baseline brain activity and functional interactions. Based on the observed activity and functional interactions, we estimated an energy landscape, searched for local minima in the energy landscape, and constructed a disconnectivity graph among the local minima.

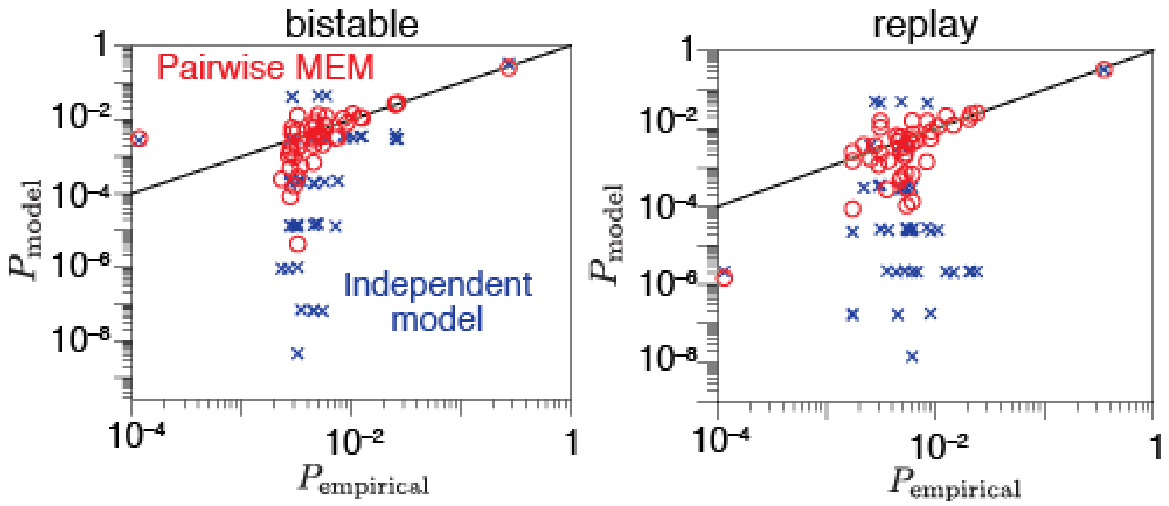

Supplementary Figure 2. Fit of the pairwise maximum entropy model to the grouped data. In both the bistable (left panel) and replay (right panel) sessions, the pairwise MEM fitted the recorded fMRI signals with a higher accuracy (more than 85%) than the independent MEM, in which J_{ij} is pinned to zero. Each symbol represents an activity pattern. Red circles and blue crosses show the results for the pairwise and independent MEMs, respectively.

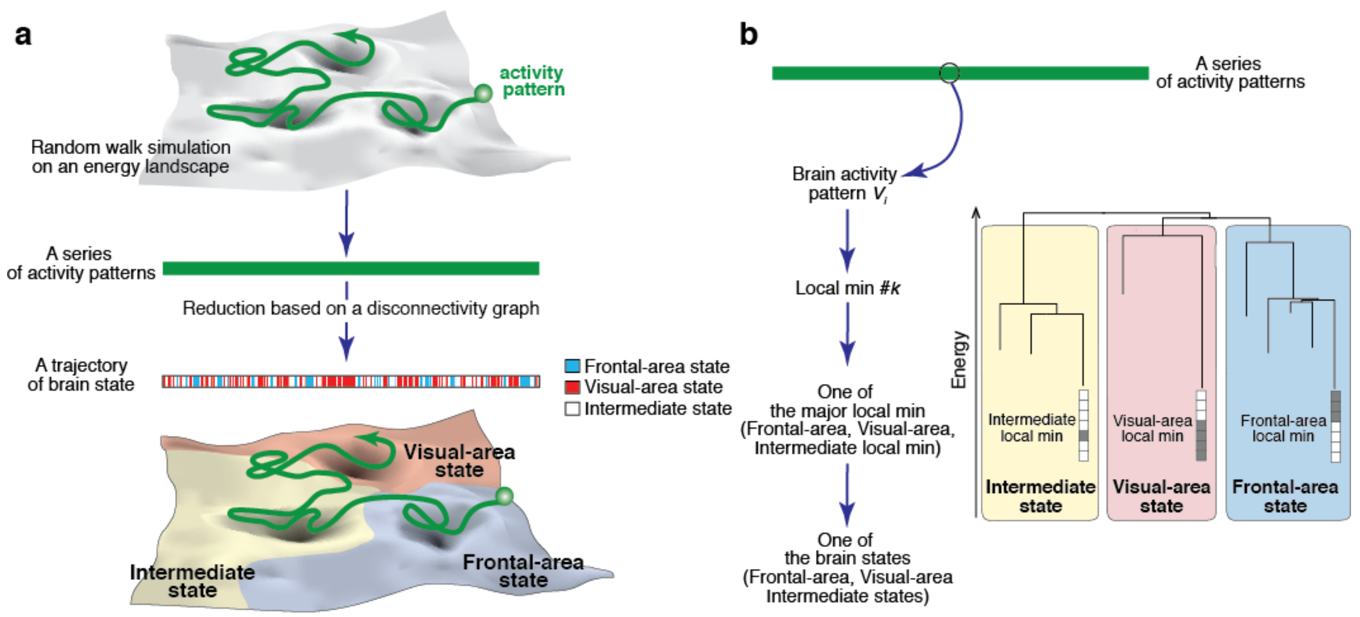

Supplementary Figure 3. Schema of the numerical simulation of the brain state dynamics on energy landscapes.

a. Overall design of the simulation. For each participant, we first numerically simulated random walks of the brain activity pattern on the estimated energy landscape by using a Markov chain Monte Carlo method with the Metropolis-Hastings algorithm. Based on the disconnectivity graph, the obtained series of activity patterns were then reduced to a (simulated) time series of stays and transitions among the brain states that we could compare to the actual reports of the participants.

b. Reduction of the series of the activity patterns. The simulated series of the brain activity patterns were reduced to a series of the brain states. First, a brain activity pattern (V_i) was reduced to one of the local minima (local min #*k*) whose basin included V_i. Second, based on the disconnectivity graph, the local minimum (#*k*) was reduced to one of the major local minima (#1 in the case of the figure), which is the same as the label of the assigned brain state (in the present case, V_i belongs to brain state #1). We carried out this procedure for all the brain activity patterns and reduced the series of the brain activity patterns to that of the brain states.

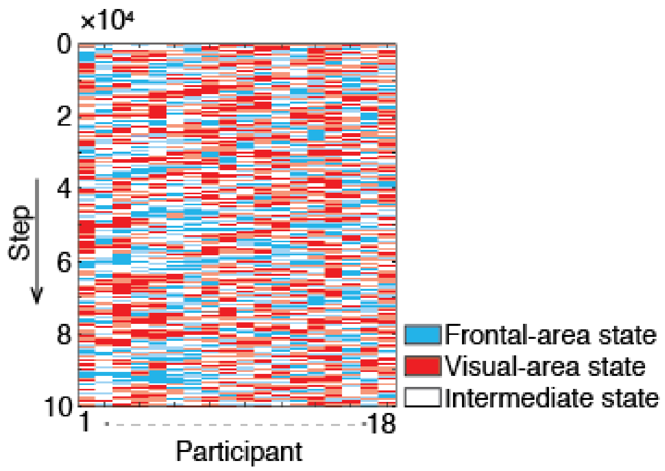

Supplementary Figure 4. Numerical results for all the participants.
Each column represents the numerically simulated trajectory of the brain state for a participant.

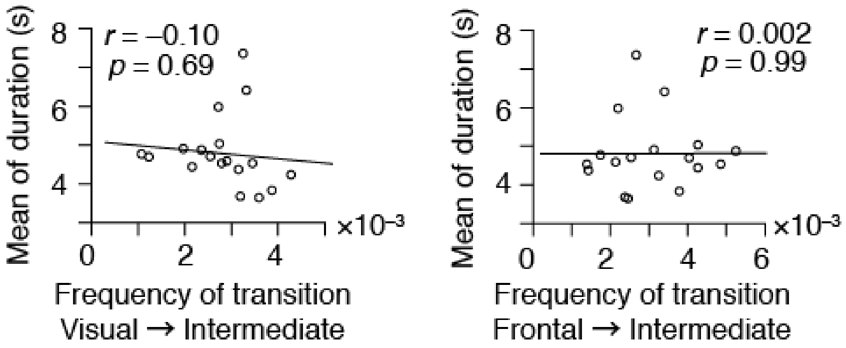

Supplementary Figure 5. Comparison of transition frequencies and behavior.
In contrast to transitions from Intermediate state to Visual-area state and from Intermediate state to Frontal-area state (Fig. 3e), we did not find significant correlations between the mean percept duration in bistable perception and the frequency of transition either from Visual-area state to Intermediate state or from Frontal-area state to Intermediate state.

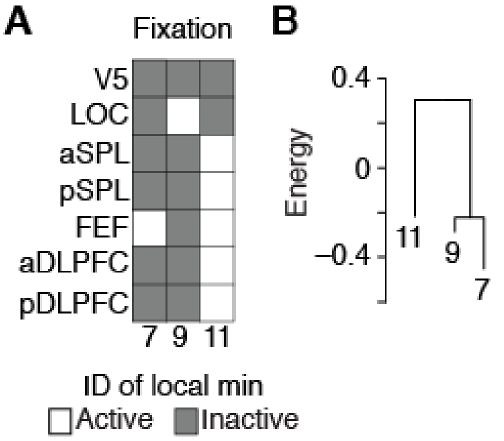

Supplementary Figure 6. Energy landscape during fixation periods.
To examine the specificity of energy landscape to cognitive statuses, we calculated an energy landscape based on brain activity during fixation periods between stimulus periods, and compared it with that during bistable perception (Fig. 1c and 1e). The fitting accuracy for the data during the fixation period (85%) was as high as that during stimulus periods. In contrast, the observed energy landscape was largely different from that during bistable perception sessions. The three major local minimums found in bistable sessions were not detected in fixation periods. Instead, the energy landscape during the fixation periods was similar to that of the fronto-parietal network during the resting state, which had been reported in our previous study[1]. This consistency might be due to the similarity between fixation and the resting state, and the fact that aSPL, pSPL, aDLPFC, and aDLPFC used in the present study are spatially close to the regions constituting the fronto-parietal network in the previous study.

Supplementary Table 1. Coordinates of regions of interest

	MNI coordinates		
Anatomical label	x	y	z
hMT/V5	47	-72	1
LOC	46	-78	2
aSPL	36	-45	44
pSPL	38	-64	32
FEF	38	0	60
aDLPFC	44	50	10
pDLPFC	48	24	9

The coordinates of hMT/V5 and LOC were based on a study by Freeman and colleagues[2]; those of aSPL and pSPL were the same as those in studies on TMS-induced effects on bistable perception[3-5]; the coordinates of FEF was based on a study by Sterzer and colleagues[6]; those of aDLPFC and pDLPFC were determined by a study by Knapen and colleagues[7] and one by Kleinschmidt and colleagues[8], respectively. MNI, Montreal Neurological Institute; see the caption of Fig. 1a for the other abbreviations.

Supplementary references

1. Watanabe, T. *et al.* Energy landscapes of resting-state brain networks. *Front Neuroinform* **8,** 12 (2014).
2. Freeman, E. D., Sterzer, P. & Driver, J. fMRI correlates of subjective reversals in ambiguous structure-from-motion. *JOV* **12,** 35–35 (2012).
3. Kanai, R., Bahrami, B. & Rees, G. Human parietal cortex structure predicts individual differences in perceptual rivalry. *Curr Biol* **20,** 1626–1630 (2010).
4. Kanai, R., Carmel, D., Bahrami, B. & Rees, G. Structural and functional fractionation of right superior parietal cortex in bistable perception. *Curr Biol* **21,** R106–7 (2011).
5. Carmel, D., Walsh, V., Lavie, N. & Rees, G. Right parietal TMS shortens dominance durations in binocular rivalry. *Curr Biol* **20,** R799–800 (2010).
6. Sterzer, P., Russ, M. O., Preibisch, C. & Kleinschmidt, A. Neural correlates of spontaneous direction reversals in ambiguous apparent visual motion. *NeuroImage* **15,** 908–916 (2002).
7. Knapen, T., Brascamp, J., Pearson, J., van Ee, R. & Blake, R. The role of frontal and parietal brain areas in bistable perception. *J Neurosci* **31,** 10293–10301 (2011).
8. Kleinschmidt, A., Büchel, C., Zeki, S. & Frackowiak, R. S. Human brain activity during spontaneously reversing perception of ambiguous figures. *Proc R Soc B* **265,** 2427–2433 (1998).
